# A Woman with Right Shoulder Pain

**DOI:** 10.5811/cpcem.7200

**Published:** 2024-06-03

**Authors:** Kitan Akinosho, William Weber

**Affiliations:** * Beth Israel Deaconess Medical Center, Department of Emergency Medicine, Boston, Massachusetts; † Rush University Medical Center, Department of Emergency Medicine, Chicago, Illinois

**Keywords:** *inferior shoulder dislocation*, *luxatio erecta*, *shoulder pain*

## Abstract

**Case Presentation:**

We report a case of an 89-year-old female who presented with pain in her right shoulder following a fall onto her outstretched hand. Upon presentation, her right hand was held behind her head and elbow held above her head in flexion. There was obvious deformity seen and felt in her axilla. Radiograph of the shoulder showed an inferior shoulder dislocation and impacted humeral neck fracture. Given her age and comorbid osteoporosis, a bedside reduction was performed by orthopedics where the humeral head was intentionally dislocated from the humeral shaft. Thirteen days after the initial shoulder dislocation, the patient’s shoulder was successfully repaired by open reduction.

**Discussion:**

Luxatio erecta, which means “erect dislocation” in Latin, refers to an inferior shoulder dislocation. It accounts for less than 1% of shoulder dislocations. Our case report highlights an inferior shoulder dislocation with a rare, concomitant humeral neck fracture, managed via staged reduction by orthopedics with intentional dislocation of the humeral head given concern over patient’s age and osteoporosis. The patient was eventually successfully repaired via arthroplasty within two weeks.

Population Health Research Capsule What do we already know about this clinical entity?

*Luxatio erecta (inferior shoulder dislocation) is a rare occurrence (<1%) that carries a high rate of concomitant vascular, neurologic, and bony injury.*

What is the major impact of the image(s)?

*We highlight a luxatio erecta with a superimposed humeral fracture, managed via bedside reduction with intentional dislocation of the humeral head prior to surgery.*

How might this improve emergency medicine practice?

*The approach to reducing luxatio erecta differs from other shoulder dislocations. Maintain high suspicion for other injuries and consider orthopedic consult.*

## CASE PRESENTATION

**Image 1.**
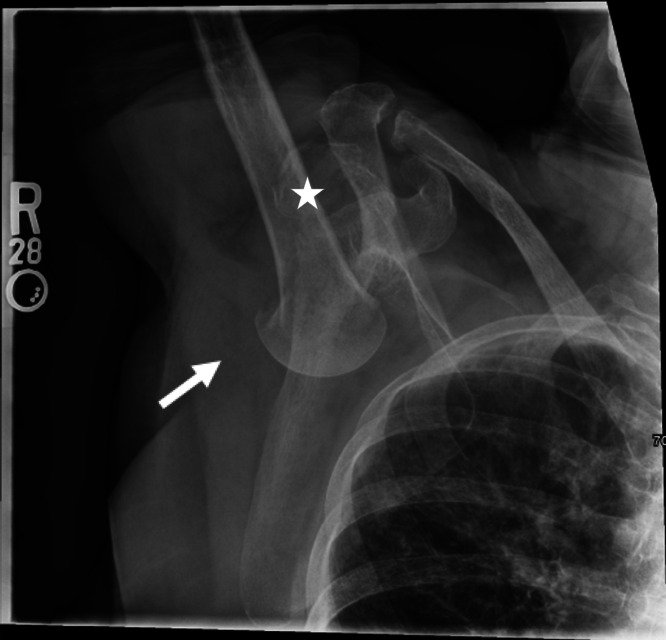
Initial radiograph of right shoulder shows a right inferior dislocation [luxatio erecta] with a concomitant impacted humeral neck fracture (arrow). A mildly displaced right glenoid fracture is also noted (star).

An 89-year-old female with a history of osteoporosis presented after a mechanical fall with right shoulder pain. She tripped and fell with her arm raised above her head and in front of her. She reported difficulty moving her right arm from its position above her head. On physical exam, her right arm was neurovascularly intact, but held above her head with palpable inferior displacement of the humeral head in the axilla. Radiograph was performed and is shown below (Image 1).

## DISCUSSION

This radiograph shows an inferior shoulder dislocation, also known as luxatio erecta, an uncommon shoulder injury that accounts for less than 1% of shoulder dislocations.^1^ Luxatio erecta typically results from sudden hyperabduction, which forces the humeral head inferiorly, often tearing the inferior capsule. Patients generally present with the arm fully abducted and their hand near their head for comfort.^2^ Patients most commonly fracture the greater tuberosity, but this patient with osteoporosis experienced a humeral neck fracture.

Two important approach considerations differentiate inferior shoulder dislocations from typical anterior or posterior dislocations: rate of concomitant injury and vector of reduction. Physicians should pay close attention for neurovascular compromise, with the axillary nerve and axillary artery very susceptible to injury. Studies have found high rates of bony injury (60%), neurologic injury (29%), and vascular injury (10%) associated with the injury.^1^ ^,^ ^3^ Reductions for inferior shoulder dislocations typically employ superior and external traction.^4^ Alternatively, anterior traction can convert an inferior dislocation to an anterior dislocation, which can then be reduced with a variety of methods.

**Image 2.**
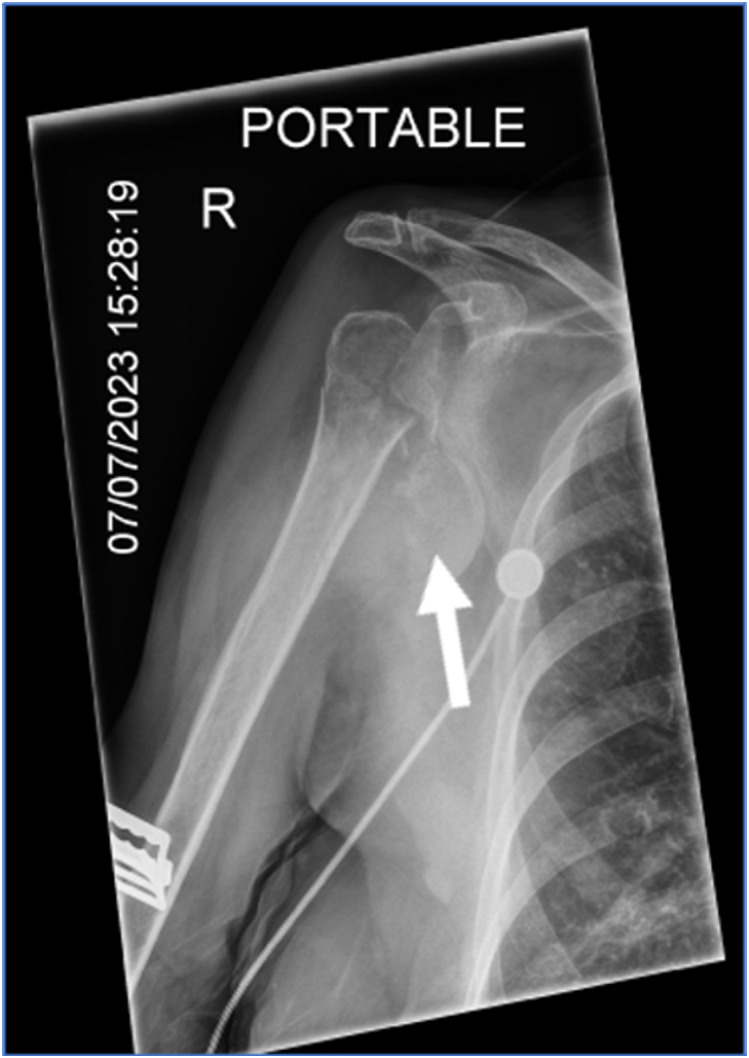
Radiograph of right shoulder immediately post-reduction showing humeral fracture (arrow).

**Image 3.**
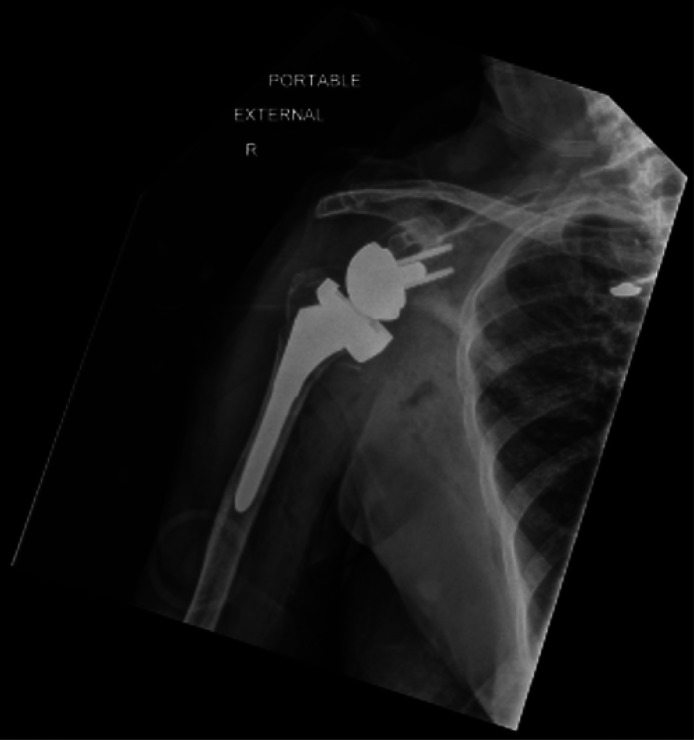
Radiograph of right shoulder 13 days post-dislocation following outpatient, right reverse total shoulder arthroplasty.

In this case, orthopedics was consulted due to the multiple concomitant fractures. Typically, the remainder of the humerus can be used to distract the humeral head and allow reduction, but in this case the humeral head was separated. In this case, orthopedics chose to use traction-countertraction with a superior and external vector away from the patient to reduce the remainder of the humerus for comfort and neurovascular safety while leaving the humeral head dislocated for open repair (Image 2). The patient was provided a shoulder sling in the interim until cardiac risk evaluation cleared her for operative repair (Image 3). The patient experienced no long-term neurologic compromise from her injury.
